# The Interaction of Hypericin with SARS-CoV-2 Reveals a Multimodal Antiviral Activity

**DOI:** 10.1021/acsami.1c22439

**Published:** 2022-03-18

**Authors:** Pietro Delcanale, Eleonora Uriati, Matteo Mariangeli, Andrea Mussini, Ana Moreno, Davide Lelli, Luigi Cavanna, Paolo Bianchini, Alberto Diaspro, Stefania Abbruzzetti, Cristiano Viappiani

**Affiliations:** † Dipartimento di Scienze Matematiche, Fisiche e Informatiche, Università degli Studi di Parma, 43124 Parma, Italy; ‡ Nanoscopy @ Istituto Italiano di Tecnologia, 16152 Genova, Italy; § Istituto Zooprofilattico Sperimentale della Lombardia e dell’Emilia Romagna, 25124 Brescia, Italy; ∥ Dipartimento di Oncologia-Ematologia, Azienda USL di Piacenza, 29121 Piacenza, Italy; ⊥ DIFILAB, Dipartimento di Fisica, Università di Genova, 16146 Genova, Italy

**Keywords:** hypericin, SARS-CoV-2, broad-spectrum antivirals, photodynamic therapy, photosensitization, photodisinfection

## Abstract

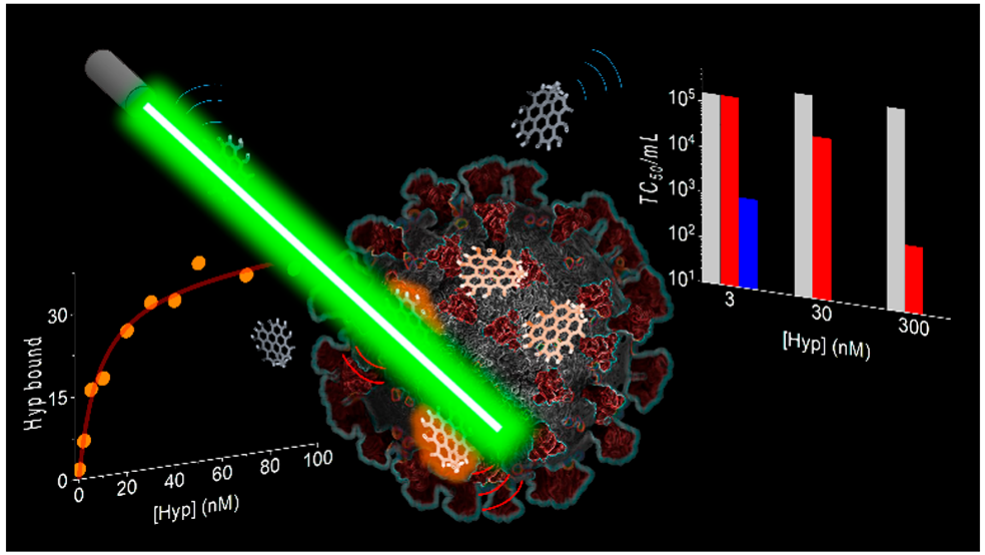

Hypericin is a photosensitizing drug that is active against membrane-enveloped viruses and therefore constitutes a promising candidate for the treatment of SARS-CoV-2 infections. The antiviral efficacy of hypericin is largely determined by its affinity toward viral components and by the number of active molecules loaded on single viruses. Here we use an experimental approach to follow the interaction of hypericin with SARS-CoV-2, and we evaluate its antiviral efficacy, both in the dark and upon photoactivation. Binding to viral particles is directly visualized with fluorescence microscopy, and a strong affinity for the viral particles, most likely for the viral envelope, is measured spectroscopically. The loading of a maximum of approximately 30 molecules per viral particle is estimated, despite with marked heterogeneity among particles. Because of this interaction, nanomolar concentrations of photoactivated hypericin substantially reduce virus infectivity on Vero E6 cells, but a partial effect is also observed in dark conditions, suggesting multiple mechanisms of action for this drug.

## Introduction

Although a number of human diseases are known to be associated with viral infections, only a handful of very specific antiviral therapies are approved, often with a narrow spectrum of coverage, which cannot provide adequate global health protection and security preparedness. In this sense, broad-spectrum drugs have been suggested as a suitable response.^1^ A number of viral pathogens causing recurrent infectious diseases are membrane-enveloped viruses, which need fusion of viral and cell membranes for virus entry. Therefore, the targeting of the viral envelope and the membrane fusion process are emerging strategies in the development of broad-spectrum antivirals,^2−5^ also based on photosensitizing compounds.^6−9^ Although photosensitization-based inactivation has been recently proposed as a strategy to tackle the current pandemic originating from the severe acute respiratory syndrome coronavirus 2 (SARS-CoV-2),^10−16^ very little experimental work has been reported so far.^17^

In this context, hypericin (Hyp) is a promising candidate. Hyp is a chromophore found in *Hypericum perforatum* plants and it is one of the most effective natural photosensitizing molecules. Hyp induces significant inactivation of several viruses upon exposure to visible light and, in some cases, also under dark conditions.^18,19^ In particular, it has been reported that Hyp inactivates a variety of enveloped viruses, while it is inactive against viruses lacking membranes.^20^ However, the efficacy of this drug is critically dependent on its aggregation state. Monomeric Hyp generates singlet oxygen (quantum yield Φ_Δ_ = 0.28 in DMSO^21,22^) and other toxic reactive oxygen species upon photoexcitation, also displaying a bright fluorescence emission (quantum yield Φ_F_ = 0.35 in DMSO^22^). Conversely, Hyp is essentially insoluble in water where it forms inactive aggregates.^23^ The photoactivity of Hyp in aqueous media is readily recovered when the molecule binds to other species that prevent its aggregation, such as membranes^24,25^ or proteins with suitable hydrophobic pockets or clefts.^26^ Hence, the affinity for phospholipidic and protein moieties is a key parameter regulating the antiviral efficacy of Hyp.

Next to binding affinity, the overall efficacy of Hyp also depends on the intrinsic heterogeneity of single viral particles, for example, in terms of size and surface accessibility, that might determine a diversified extent of drug loading. In this regard, experimental approaches addressing the behavior of single viral particles, overcoming averaged information, are recently getting increasing attention for the improvement of antiviral drugs.^27,28^

Despite the antiviral treatments based on Hyp were proposed long ago,^9^ quantitative measurements addressing binding affinity and loading on single viral particles are lacking. This suggests a renewed potential for Hyp-based antiviral applications, which can provide broad-spectrum action against membrane-enveloped viruses, including SARS-CoV-2.

In this work, we use a combination of fluorescence microscopy, spectroscopy, and viral assays to demonstrate that Hyp binds to the membrane envelope of SARS-CoV-2 and significantly inactivates the virus, both upon photoactivation and in the dark. The experiments are mainly focused on providing quantitative measurements of Hyp affinity for SARS-CoV-2 and its loading on viruses, also at the level of individual viral particles. A first evaluation of the antiviral efficacy of Hyp is also shown, and possible mechanisms of action are discussed.

## Experimental Section

### Materials

Hypericin was obtained from HWI pharma services GmbH and dissolved in DMSO to obtain a concentrated stock solution. Concentration was measured spectroscopically. Syto13 was obtained from ThermoFisher Scientific and dissolved in PBS buffer pH = 7.4 to obtain a stock solution (250 nM). Recombinant soluble Receptor Binding Domain of the SARS-CoV-2 spike protein was obtained from Sino Biological, dissolved as indicated by the provider to obtain a stock concentration of 7 μM that was stored at −20 °C. The recombinant protein is expressed with a His-tag and is biotinylated (2.2 biotins per protein). All samples were store in the dark and used fresh, avoiding repeated freeze–thaw cycles.

### Instrumentation

#### Microscopy

**Figure 1:**
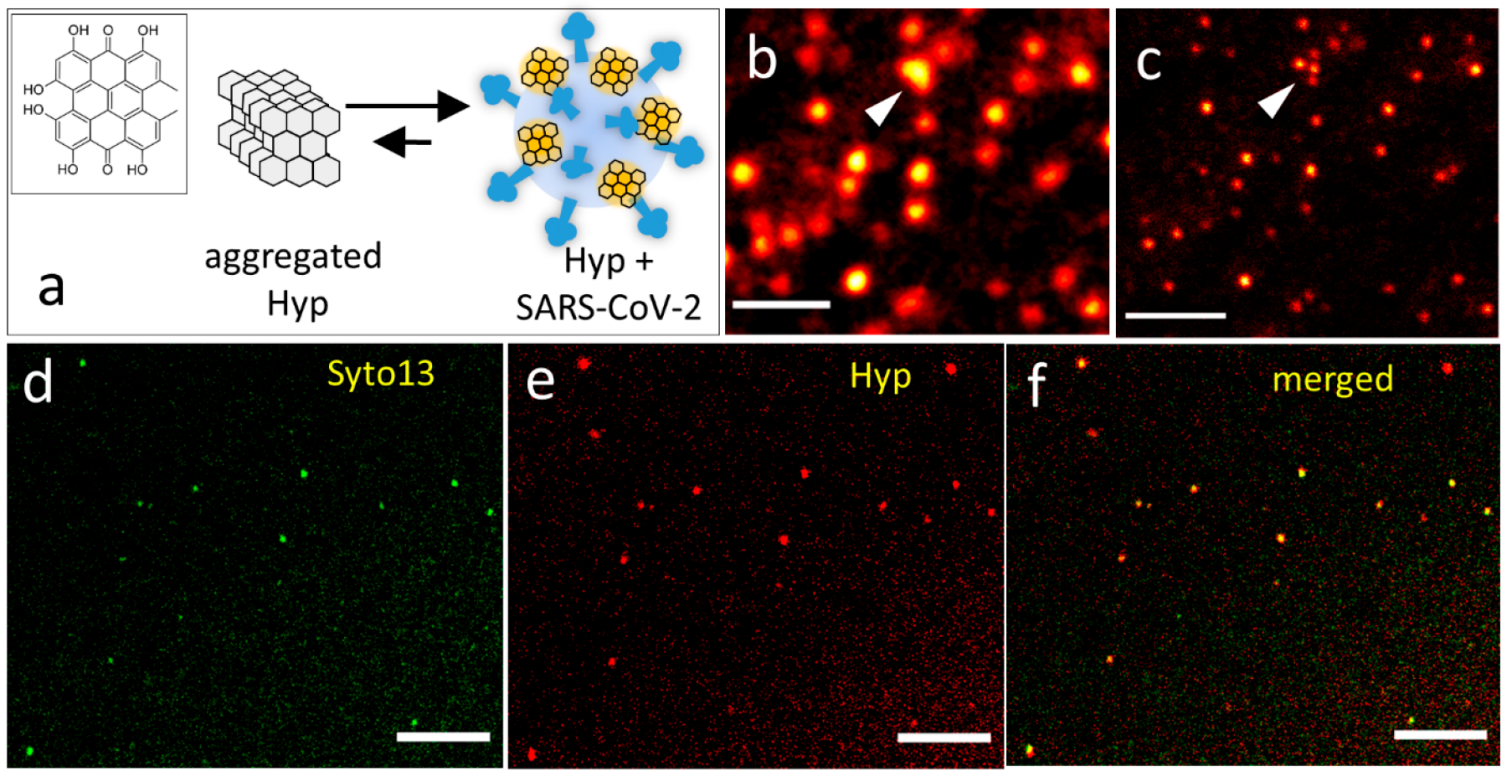
(a) Schematic representation of the interaction between Hyp and SARS-CoV-2 in aqueous solution: aggregated Hyp (left) is not fluorescent, while Hyp bound to SARS-CoV-2 particles (right) is fluorescent. The inset shows the chemical structure of Hyp. (b,c) Confocal (b) and corresponding STED (c) images of SARS-CoV-2 particles exposed to Hyp (red). The arrows indicate a cluster of viral particles that is well-resolved by STED imaging. Scale bars 500 nm. (d–f) Confocal images of the same field of view showing SARS-CoV-2 particles labeled with the RNA probe Syto13 and exposed to Hyp. Syto13 (d, green) and Hyp (e, red) channels are separately shown, together with the corresponding two-color image (f), where the overlap of the two colors looks yellow. Scale bars 5 μm.

Colocalization (Figure 1d–f) and single-particle intensity (Figure 3) measurements were obtained with a Nikon TiE inverted confocal microscope equipped with three excitation lasers (405, 488, and 561 nm) and three alkaline PMTs. Hyp and Syto13 were excited at 561 and 488 nm, respectively. The 561 nm laser was a CW diode pump solid-state laser (Melles Griot), while the 488 nm laser was a CW diode semiconductor laser (Coherent). Fluorescence emission was collected through two bandpass filters (Hyp, 605/70 nm and Syto13, 515/30 nm).

STED nanoscopy (Figure 1b,c) was performed using the Leica Microsystems STELLARIS 8 STED equipped with a supercontinuum White Light Laser (WLL) where the 561 nm excitation wavelength was selected by an AOBS. The depletion wavelength was 775 nm.

#### Fluorescence Correlation Spectroscopy

Fluorescence correlation spectroscopy (FCS) experiments were performed using a Microtime 200 system from PicoQuant, based on an inverted confocal microscope (Olympus IX71) and equipped with two SPADs (Single Photon Avalanche Diodes) used in cross-correlation mode. Hyp excitation was achieved by a 475 nm picosecond diode laser operated at 20 MHz. Fluorescence emission was collected through a bandpass filter (675/25 nm) and split with a 50/50 splitter between the two detection channels. The setup allowed the simultaneous acquisitions of correlation curves and time-resolved fluorescence decays, measured by time-correlated single photon counting (TCSPC).

#### Spectroscopy

Absorption spectra were collected using a Jasco V-650 (Jasco Europe) spectrophotometer. Steady-state fluorescence excitation, emission, and anisotropy spectra were measured with a SF5 spectrofluorometer (Edinburgh Instruments Ltd., Livingston, U.K.). The instrument is equipped with excitation and emission polarizers, enabling fluorescence anisotropy detection.

### Sample Preparation, Acquisition, and Analysis

#### Fluorescence Spectroscopy, FCS, and Lifetimes

**Figure 2:**
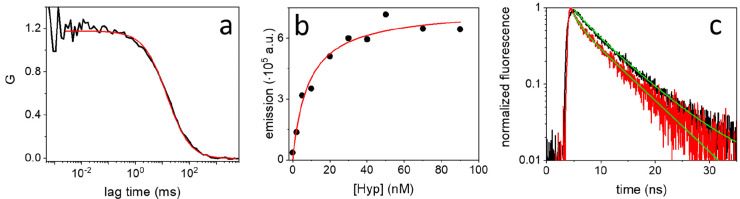
(a) Representative correlation curve measured by FCS (black) on a solution containing SARS-CoV-2 particles exposed to 5 nM Hyp. The red line shows the results of the fitting with a model comprising a single diffusing species. (b) Integrated fluorescence emission of Hyp, at increasing concentration, in the presence of the same amount of SARS-CoV-2 particles (black circles). The red line shows the result of the fitting with a binding model. (c) Normalized time-resolved fluorescence decays observed for Hyp bound to DLPC liposomes (black) and SARS-CoV-2 particles (red). The green lines show the results of the fitting with a biexponential model for both decays.

The stock suspension of fixed viral particles was preliminarily centrifuged (3 min, 5000 rpm) to remove the largest aggregates. Viral particles were then diluted 50 times in PBS buffer pH = 7.4, and a small volume of concentrated Hyp in DMSO (50× the desired final concentration) was added. The final concentration of DMSO was 2%. After an incubation of 5 min at room temperature in the dark, the solution (50 μL) was placed in the fluorometer using a quartz cuvette (*l* = 0.3 cm). Emission spectra (Figures 2b and S3) were acquired by exciting the sample at 553 nm, while excitation and anisotropy spectra (Figure S3) were acquired by collecting the emission at 650 nm. Directly after, ∼40 μL of the same solution was placed on the FCS system for the simultaneous measurement of correlation curves (Figures 2a and S5) and time-resolved fluorescence (Figures 2c and S6c), with an acquisition time of 5 min, and 3–5 repetitions for each sample. All samples were prepared in the same way and measured under the same conditions. The low concentrations ensured that measurements are not affected by any artifacts related to the optical density of samples at the excitation wavelength. The fitting of measured correlation curves (Figures 2a, S5, S7) and fluorescence decays (Figures 2c, S6) were performed with the SymphoTime software from PicoQuant. Decays were well-fitted by a biexponential model while correlation curves were well-fitted using a model comprising a single type of diffusing species, according to the equation:
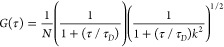
where τ is the lag time, τ_D_ is the diffusion time of the species through the detection volume, *N* is the average number of diffusing species in the detection volume, and *k* is an instrumental parameter related to the detection volume. Because the detection volume of the instrument is known, the three-dimensional diffusion coefficient and the average concentration of the diffusing species are calculated by the software from the values of τ_D_ and *N* found in the fitting.

DLPC liposomes were prepared according to the injection method. DLPC (7 mg/mL) dissolved in ethanol (700 μL) was slowly injected into 10 mL of a 10 mM phosphate buffer, pH 7. The solution was kept at 30 °C and magnetically stirred. Liposomes were exposed to Hyp as described above for viral particles and were placed on the FCS system for measurement of time-resolved fluorescence (Figure 2c).

#### Imaging

The stock suspension of fixed viral particles was preliminarily centrifuged (3 min, 5000 rpm) to remove the largest aggregates and viral particles were diluted 50 times in PBS buffer pH = 7.4.

**Figure 3:**
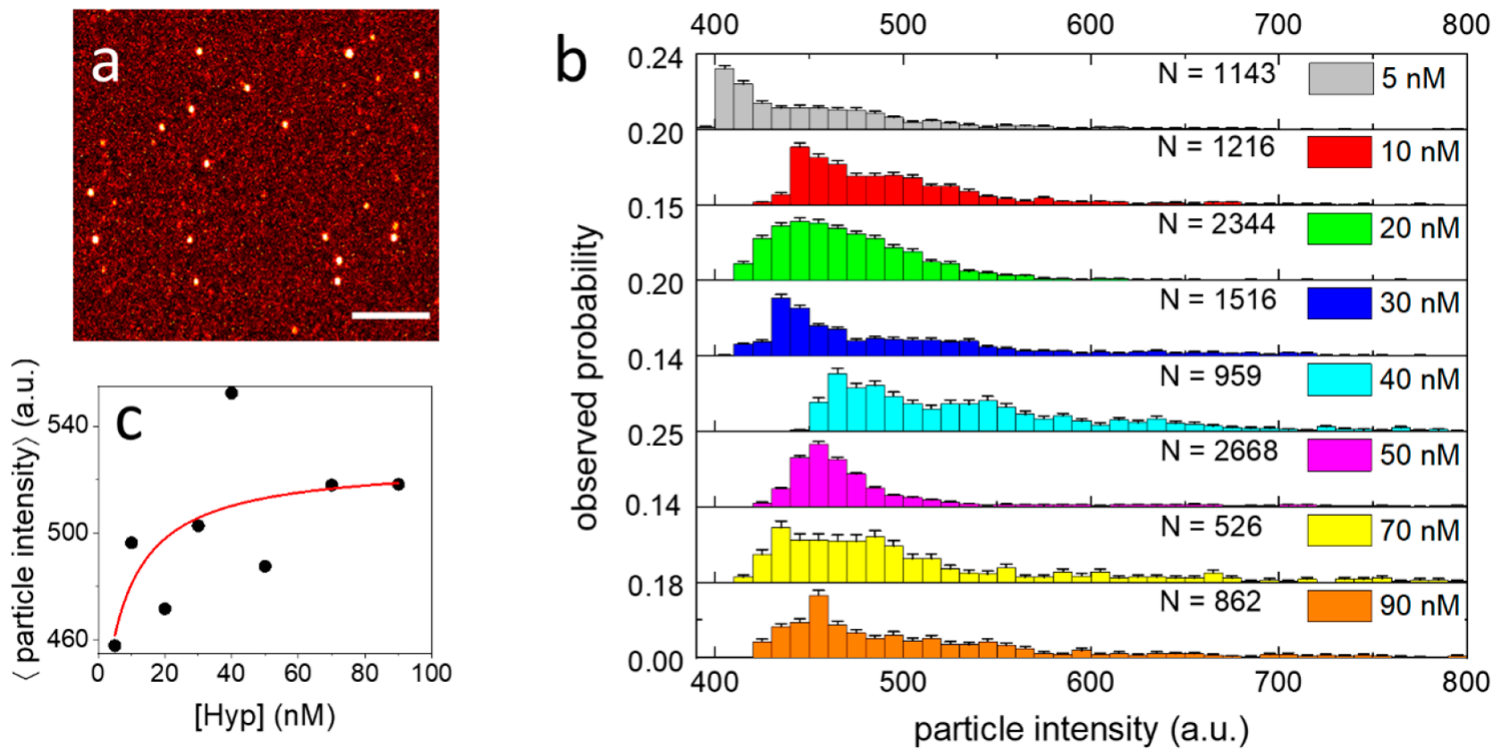
(a) Representative image of SARS-CoV-2 particles exposed to Hyp, acquired for the analysis of single-particle intensity. Scale bar 5 μm. (b) Observed probability distributions of single viral particle fluorescence intensity obtained for the same amount of SARS-CoV-2 exposed to 5 nM (gray), 10 nM (red), 20 nM (green), 30 nM (blue), 40 nM (cyan), 50 nM (magenta), 70 nM (yellow), 90 nM Hyp (orange). *N* is the number of analyzed viral particles for each distribution. Bin width = 10 au (c) Average values of single viral particle fluorescence intensity measured at increasing Hyp concentration. The red line shows the result of the fitting with a binding model. Reported Hyp concentrations refer to the incubation with SARS-CoV-2.

For single-particle intensity measurements (Figure 3), prediluted viral particles were mixed with Hyp (concentrations: 5, 10, 20, 30, 40, 50, 70, 90 nM), and 20 μL of each solution was seeded on a sterile cell culture dish with glass bottom. After a 10 min incubation, 100 μL of PBS was added, without intermediate washings, to reach the optimal volume for imaging, which allows complete and homogeneous coverage of glass with the solution. The sample was finally sealed to avoid evaporation and imaged immediately. The very same procedure was followed for all the solutions to enable comparison.

For colocalization measurements (Figure 1d–f), prediluted viral particles were mixed with Hyp (final concentration: 50 nM), seeded on sterile cell culture dish and subsequently mixed with Syto13 (final concentration: 50 nM). The sample was then imaged immediately without intermediate washings. A sequential scanning mode was used to exclude bleed-through during the acquisition procedure.

In all the samples, the final concentration of DMSO is negligible (<2%).

#### Single-Particle Intensity Analysis

Single-particle intensity data of Figure 3 and Figure S8 were acquired using the same parameters: 1024 × 1024 image size, pixel dwell time 6.2 μs, pixel size 80 nm, line average × 4; and with comparable axial position of the focal plane. Acquired images (8–10 for each sample) were analyzed using ImageJ. First, a Gaussian filter (σ = 180 nm) was applied to reduce uncorrelated background noise and better discriminate fluorescent particle. In a few cases, parts of the images that resulted clearly out of focus, e.g. due to irregularities of the glass surface, were excluded. Then, a particle analyzer tool was applied to automatically recognize particles having minimum intensity above a threshold value (400–420 au) and a minimum area (8–10 pixels). Results of the analysis were visually inspected and a few particles (<1%) showing unrealistic features, e.g. corresponding to aggregates, were excluded. Finally, the mean intensity values of each selected particle were stored and used to reconstruct distributions.

#### Negative Staining Electron Microscopy (nsEM)

The nsEM imaging (Figure S2) was carried out on the resuspended pellet of fixed viral particles from ultracentrifugation by using the “Airfuge” method. This consists in the ultracentrifugation of 85 μL of a dilution 1:5 in PBS of the pelleted materials in Airfuge Beckman (Airfuge, Beckman Coulter Inc. Life Sciences, Indianapolis, Indiana, U.S.A.) for 15 min at 21 psi (82 000*g*).^29^ The grids were stained using 2% sodium phosphotungstate (NaPt), pH 6.8, for 1.5 min, and observed with a Tecnai G2 Spirit BioTwin transmission electron microscope (FEI, Hillsboro, OR, U.S.A.) operating at 85 kV.

### Viral Infectivity Assays and Virus Fixation

Isolation of SARS-CoV-2 virus as well as all the further experiments to evaluate the antiviral efficacy of Hyp against SARS-CoV-2 infected cells were performed in biosafety level 3 (BSL3) laboratories. SARS-CoV-2 virus isolation was obtained through inoculation of infected biological human sample in Vero E6 cell lines. SARS-CoV-2 HCoV-19/Italy/310902/46/2020 strain (GISAID code: EPI_ISL_9011947), belonging to clade 20A (Nextstrain naming), was propagated in the same cell line and incubated at 37 °C with 5% CO_2_. Viral titer (TCID_50_/mL) was verified by Reed-Muench assay.

The antiviral efficacy of Hyp was investigated by using different Hyp concentrations obtained by dilution of Hyp in DMSO. Three experiments were carried out at 300, 30, and 3 nM of Hyp. For each Hyp concentration tested, five samples were prepared: *SARS-CoV-2 + Hyp light*; *SARS-CoV-2 + Hyp dark*; *SARS-CoV-2*; *Hyp*; *DMSO*.

First, 900 μL of viral suspension was put in contact with 100 μL of Hyp to obtain the desired final concentration of Hyp. One aliquot of this mixture was subjected to lamp illumination for 15 min with an intensity of 22 mW/cm^2^, corresponding to a fluence of 20 J/cm^2^ (*SARS-CoV-2 + Hyp light*). The other aliquot of the same mixture was kept in the dark for the same time and was used to evaluate dark effects (*SARS-CoV-2 + Hyp dark*).

Two other samples consisting of 900 μL of Dulbecco’s Modified Eagle Medium (DMEM) with 100 μL of Hyp (*Hyp*) and 900 μL of DMEM with 100 μL of DMSO (*DMSO*) were included as Hyp and DMSO controls, respectively, to evaluate the possible presence of the cytotoxic effect of these substances on the Vero E6 cell line. Finally, the virus reference sample consisting of 900 μL virus with 100 μL DMEM (*SARS-CoV-2*) was included to verify the actual titer of the virus used in this experiment.

Samples *SARS-CoV-2 + Hyp light*, *SARS-CoV-2 + Hyp dark* and *SARS-CoV-2* were inoculated into 96-well plates of preformed monolayer Vero E6 cells (>85%) in 10-log dilutions from dilution −1 to −8, and the virus titer was calculated using the Reed and Muench method and expressed as TCID_50_/mL. Control samples *Hyp* and *DMSO* were inoculated into 24-well plates of Vero E6 cells to assess the possible presence of toxicity of Hyp and DMSO on Vero E6 cells.

To obtain fixed SARS-CoV-2 virions, the culture medium from infected Vero E6 cells, inoculated as reported above, was collected at 72 h after infection, clarified by centrifugation at 3200*g* for 20 min, and fixed with 4% formaldehyde for 30 min at room temperature. SARS-CoV-2 virions inactivated by fixation were concentrated from the medium by ultracentrifugation through a 20% (w/w) sucrose cushion (120 min at 147 000*g* in a Beckman TY50.2 Ti rotor; Beckman Coulter Life Sciences). Pelleted particles were resuspended in PBS and stored in aliquots at −80 °C.

## Results and Discussion

### Binding of Hyp to SARS-CoV-2

From a structural point of view, Hyp (Figure 1a, inset) has a hydrophobic core, which is responsible for the extended aggregation in aqueous solvents. Even if aggregated Hyp absorbs visible photons, molecular excited singlet and triplet states are rapidly quenched resulting in negligible fluorescence emission and undetectable generation of reactive oxygen species, that is, Hyp is inactive from a photophysics point of view (Figure S1). We thus investigated the effect of SARS-CoV-2 particles on Hyp fluorescence emission. To this end, viral particles were preliminarily fixed with formaldehyde, washed, and concentrated by ultracentrifugation, and their overall integrity was qualitatively checked by electron microscopy (Figure S2). Figure 1a represents the interaction of Hyp molecules with SARS-CoV-2 particles in aqueous environment: the presence of viral particles solubilizes Hyp aggregates, so that virus-bound Hyp molecules recover a significant fluorescence emission upon photoexcitation, as qualitatively confirmed by fluorescence spectroscopy measurements (Figure S3a). In addition, the high fluorescence anisotropy of Hyp exposed to SARS-CoV-2 particles (Figure S3b) indicates that rotational averaging is prevented, as expected for a fluorophore bound to a bulky viral particle. Fluorescence emission was then exploited to directly visualize the loading of Hyp on SARS-CoV-2 particles with fluorescence microscopy.

In a first imaging experiment, previously fixed SARS-CoV-2 particles were incubated with Hyp in aqueous buffer, then seeded on a glass coverslip and imaged with a confocal microscope. Figure 1b provides a confocal image where it is clearly visible how Hyp emission concentrates in bright fluorescent spots of diffraction limited size, compatible with SARS-CoV-2 particles (see Figure S4 for additional images). Because of the limited spatial resolution of a confocal setup, Hyp-loaded particles appear as blurred fluorescent spots, and it is not possible to clearly assess if they are isolated or organized into small clusters. Notably, Hyp can be localized in biological samples with subdiffraction resolution using stimulated emission depletion (STED) microscopy.^22,30^ Therefore, we applied STED microscopy to directly visualize bound Hyp at higher spatial resolution. Panels b and c of Figure 1 show images of the same field of view obtained with confocal and STED microscopy, respectively. The increased resolution of STED allows a clear discrimination of single viral particles, also when they are found in small clusters, even if the majority of detected particles were isolated.

In order to demonstrate that Hyp binds to intact virions, containing RNA, we performed a second imaging experiment. In this case, previously fixed SARS-CoV-2 particles were stained with Syto13, a probe that labels nucleic acids, and exposed to Hyp. Particles were then seeded on a glass coverslip and imaged with a confocal microscope. Because Hyp and Syto13 have spectrally separated emission, they are simultaneously localized in the same field of view. An example is offered in Figure 1d–f where emission of Hyp and Syto13 are in red and green, respectively. Both Syto13 (Figure 1d) and Hyp (Figure 1e) emission concentrates in bright spots of diffraction limited size, and remarkably, the large majority (∼80%) of such spots colocalizes in the two detection channels (Figure 1f). This observation confirms that Hyp is mostly bound to intact SARS-CoV-2 particles that contain RNA. The fact that a minor fraction of fluorescent particles does not show colocalization is likely due to an incomplete staining of viral RNA with Syto13, even if the presence of some debris or a few damaged particles cannot be excluded.

### Hyp Affinity for the Viral Membrane

In order to further investigate the interaction between Hyp and SARS-CoV-2, we applied fluorescence correlation spectroscopy (FCS) on SARS-CoV-2 particles exposed to Hyp, between 1 and 100 nM. FCS measures the concentration of fluorescent species in a solution and their three-dimensional diffusion coefficient (see Experimental section). Because unbound Hyp is not fluorescent, the detected diffusing species coincide with viral particles loaded with Hyp. A representative correlation curve measured by FCS is reported in Figure 2a, while others are displayed in Figure S5. The fitting of correlation curves with a model comprising a single type of diffusing species confirmed the presence of slow-diffusing particles (average diffusion coefficient *D* = (2.4 ± 1.2) μm^2^/s, see Figure S5) with a hydrodynamic diameter of ∼150 nm, in line with the expected size of SARS-CoV-2 (see Figure S2). In addition, the concentration of Hyp-loaded viral particles was calculated from the amplitude of correlation curves and was determined to be ∼1 nM, with a rather high variability (see Figure S5). This variability was attributed to the presence of rare, yet hard-to-remove, species having slower diffusion (e.g., aggregates or residual debris), affecting the calculated value.

The same solutions were also placed in a fluorometer to measure the ensemble fluorescence emission. Figure 2b shows the total emission (black circles), obtained by integration of the whole emission spectrum, measured at increasing concentration of Hyp (0–90 nM) and constant concentration of SARS-CoV-2 particles (∼1 nM, as obtained by FCS). The emission displays a steep increase between 0 and 20 nM of Hyp and reaches a saturation value around 30–40 nM. Above this concentration, SARS-CoV-2 particles cannot accommodate further Hyp so that molecules in excess form nonemissive aggregates. Because the concentration of SARS-CoV-2 particles is known by FCS, we could estimate the molar ratio of saturation, which roughly corresponds to 30:1 (Hyp: SARS-CoV-2). Moreover, the affinity of Hyp for SARS-CoV-2 particles is quantified by fitting the data with a previously determined equation^31^ (Figure 2b, red). An apparent equilibrium dissociation constant *K*_D_ = 8.5 nM is obtained at this concentration of virus.

Membranes are generally recognized as the main target of Hyp.^20^ To experimentally validate this hypothesis also for SARS-CoV-2, we analyzed time-resolved fluorescence decays of virus-bound Hyp, which provide indications about the local environment of the molecule. Figure 2c displays the normalized fluorescence decays measured for 5 nM Hyp bound to ∼1 nM SARS-CoV-2 particles (red) and 10 nM Hyp bound to ∼2 nM DLPC liposomes (black), which are used as a reference model for the membrane environment. The embedding of Hyp into liposomal membranes was directly assessed by fluorescence microscopy (Figure S6a,b). At the employed concentrations, well-below the saturation condition, bound Hyp molecules are locally isolated, and interactions with other Hyp molecules bound to the same particle, which might affect the fluorescence decay, are negligible (see Figure S6c). Qualitatively, the curves reported in Figure 2c are very similar, except for a difference in the first part of the decays. Both curves are best fitted with a biexponential model yielding two lifetimes: τ_1_ = (1.2 ± 0.1) ns, τ_2_ = (6.3 ± 0.3) ns (amplitudes 25% and 75%, respectively) for Hyp bound to SARS-CoV-2; τ_1_ = (2.3 ± 0.5) ns, τ_2_ = (6.8 ± 0.4) ns (amplitudes 10% and 90%, respectively) for Hyp bound to liposomes. In agreement with qualitative observation, the longer lifetimes (τ_2_) show consistent values, while the shorter lifetimes (τ_1_) are different. The longer lifetime components are associated with a major population of Hyp molecules found in a microenvironment less polar than water (for comparison, τ = 5.5 ns in DMSO and τ = 6.0 ns in acetone^32^), which is very similar for both liposomes and viruses, and it is reasonably identified in the phospholipidic bilayer. The shorter lifetime components are more likely associated with molecules with a higher degree of exposure to the aqueous environment.

Next to the phospholipidic bilayer, Hyp might also bind to the spike proteins that are protruding on the surface of SARS-CoV-2 particles, and in particular with the exposed receptor binding domain (RBD) of the spike protein, responsible for the binding of SARS-CoV-2 to the angiotensin converting enzyme-2 (ACE2) receptor on human cells.^33,34^ We then employed a recombinant soluble version of RBD to investigate if Hyp can specifically bind to this protein component. FCS measurements performed on Hyp exposed to RBD clearly show that Hyp does not bind monomeric RBD in the nanomolar concentration range. The same experiments reveal that Hyp binds with high affinity (*K*_D_ ∼ 60 nM) to very large aggregates of the recombinant RBD present at low concentration, which do not correspond to the biologically relevant monomeric state of this proteins (see Figure S7 for a detailed discussion). These results indicate the lack of a specific binding between Hyp and the native RBD. Nevertheless, the presence of low-affinity interactions (e.g., with micromolar *K*_D_), which are not well discriminated by FCS, cannot be excluded.

Similarly, it cannot be fully excluded that Hyp interacts with proteins of the viral capsid, located below the membrane envelope of viruses. Still, at the employed concentrations, it is unlikely that Hyp can reach such unexposed proteins in significant amounts because of the known high affinity of Hyp for the envelope membrane. While capsid proteins could be exposed through a permeabilization of the viral envelope, this procedure compromises the colloidal stability and the structural integrity of the viral particles, precluding a selective investigation of this interaction.

Altogether, the above observations strongly support the idea that the viral envelope is the primary component targeted by Hyp, even though the presence of other binding sites on viral proteins, characterized by a lower affinity, could not be completely ruled out.

### Hyp Distribution on SARS-CoV-2 Particles

Because fluorescence emission occurs from virus-bound Hyp, the emission intensity from a single viral particle is approximately proportional to the number of bound Hyp molecules. Therefore, relative differences in Hyp loading on single SARS-CoV-2 particles are easily obtained with fluorescence imaging. To do that, viral particles (∼1 nM) were first incubated with Hyp (5–90 nM) and then seeded on a glass coverslip, without washings. Prior to imaging, a constant amount of PBS buffer was added to reach a sample volume allowing complete coverage of the glass surface and optimal visualization conditions, while preserving the Hyp:SARS-CoV-2 ratio. A representative image is displayed in Figure 3a. Images were then analyzed to extract the emission intensity from single viral particles. Briefly, a threshold value is set to localize bright spots in the images, corresponding to particles displaying an intensity above the background level due to detector noise and residual glass-sticking Hyp (Figure S8). Then, the intensities of each selected particle are binned to obtain the probability distributions of Figure 3b.

All the distributions show a majority of viral particles having intensity between 430 and 500 au, with a tail at higher intensity values. It should be considered that particles with an intensity below ∼400 au could not be discriminated from the background (Figure S8), while dim particles (400–430 au), whose intensity is just above threshold, are more subject to image-to-image variability. Overall, the observed distributions (Figure 3b) do not present a well-defined shape, making it difficult to identify a clear trend. These data indicate that the loading of Hyp onto single viral particles is heterogeneous. Such heterogeneity is necessarily related to the stochastic nature of binding, but it is possibly enhanced by intrinsic differences among individual particles (e.g., in the extent of accessible membrane surface).

The approach used for sampling single viral particles is straightforward but might suffer from sensitivity limitations at very low Hyp concentrations (<10 nM). At such low amounts of Hyp, single-particle emission originates from a few molecules and, additionally, a fraction of viral particles is likely to remain unlabeled and thereby invisible to fluorescence imaging. A more systematic sampling would, then, require more complex experimental approaches, like correlative light and electron microscopy, to detect unlabeled or weakly emitting particles.^35^ Nevertheless, a trend can be identified by looking at the average values of single-particle intensity, calculated from distributions (Figure 3c). These qualitatively show an increase of intensity at growing Hyp concentration. Moreover, in spite of the high variability, a fitting of these data with the same model used in Figure 2b yields an apparent *K*_D_ = 7 nM (Figure 3c, red), in line with the value obtained from bulk measurements. It is important to point out how this type of information regarding drug loading at the level of single viral particles is inaccessible to ensemble measurements.

### Efficacy of Hyp against SARS-CoV-2

**Figure 4:**
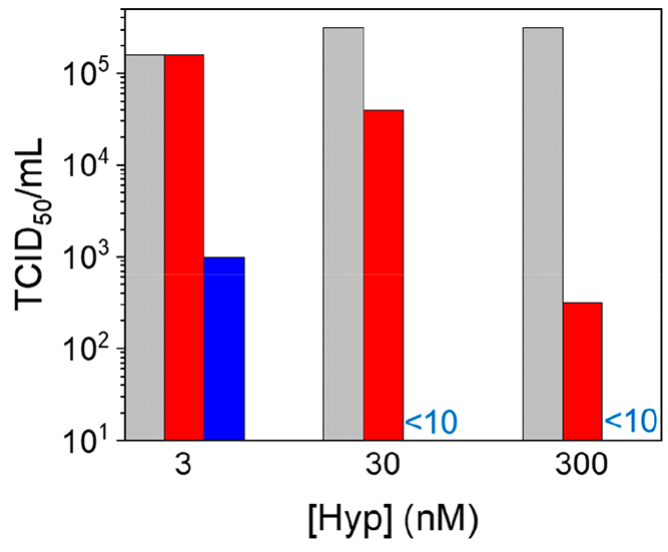
Viral titer (TCID_50_/mL) on Vero E6 cell infected with SARS-CoV-2 viruses previously exposed to increasing Hyp concentration and kept in dark (red) or irradiated with 20 J/cm^2^ blue light (blue). Reference TCID_50_/mL values obtained on Vero E6 cell infected with SARS-CoV-2 not exposed to Hyp are in gray.

Viral infectivity assays were performed to test the efficacy of Hyp against SARS-CoV-2. In such experiments, active SARS-CoV-2 particles were first incubated with Hyp (3, 30, and 300 nM). Then, samples were either kept in the dark or irradiated with blue light, to photoexcite Hyp, allowing the discrimination of both dark and photoinduced effects. After the treatment, viral samples were serially diluted and incubated with a monolayer of Vero E6 cells, while keeping dark conditions, in order to determine the residual infectivity. The 50% tissue culture infective dose per mL (TCID_50_/mL), that is, the viral titer, corresponding to the dilution at which cell viability is reduced by 50%, was then measured. Figure 4 summarizes the measured TCID_50_/mL values obtained for viruses incubated with Hyp and kept in the dark (red bars) or photoexcited (blue bars). Reference values (TCID_50_/mL ∼ 10^5^, gray bars) were obtained for fully infective viral samples, which were not treated with Hyp but were directly incubated with Vero E6 cells after serial dilution.

Under the employed conditions, a dramatic reduction of infectivity (TCID_50_/mL from 10^5.5^ to <10^1^) was observed for SARS-CoV-2 exposed to both 300 and 30 nM Hyp and photoirradiated. A clear antiviral effect was observed even at 3 nM Hyp, upon light exposure, inducing a 2.2-log reduction of the viral titer (TCID_50_/mL from 10^5.2^ to 10^3^) with respect to untreated viruses. These results demonstrate that even low concentrations of photoexcited Hyp are effective against SARS-CoV-2, consistently with the observed high affinity of Hyp toward viral particles. Our findings can be compared with recently reported photoinactivation studies on SAR-CoV-2 using methylene blue and radachlorin as photosensitizing molecules.^17^ The much lower photosensitizer concentrations employed in the present study demonstrate the higher efficacy of Hyp in SARS-CoV-2 photoinactivation and opens encouraging perspectives for the use of the compound as an effective antiviral agent.

An antiviral activity was also observed under dark conditions, even if to a lesser extent than in photoexcited samples. In the dark, infectivity reduction was nil for viruses exposed to 3 nM Hyp (TCID_50_/mL 10^5.5^) and moderate for viruses exposed to 30 nM Hyp (TCID_50_/mL from 10^5.5^ to 10^4.6^), while it became significant at 300 nM Hyp, which induced a 3-log reduction of TCID_50_/mL value (from 10^5.5^ to10^2.5^) in comparison to untreated viruses. Importantly, no toxicity was observed in control experiments carried out on Vero E6 cells exposed to Hyp or DMSO, in absence of viruses and in the dark, confirming that cells are not directly damaged by Hyp or by the small amount of DMSO added to deliver Hyp (Figure S9).

These findings highlight a multimodal activity of Hyp as antiviral agent, which reconcile previously reported controversial literature.^20^ Photoexcitation of virus-bound Hyp is likely to result in an extensive damage of the viral particles, caused by reactive oxygen species, as observed in similar studies.^6,8^ Because a single molecule of Hyp undergoes many cycles of photon absorption during a treatment, this mechanism only requires a few bound Hyp molecules per viral particle. However, such photoreaction cannot occur in the dark, suggesting the presence of a complementary mechanism, responsible for the reduction of viral infectivity in absence of light exposure. This requires a more extensive loading of Hyp on viral particles and becomes thereby significant at higher Hyp concentrations. A possibility is that the embedding of Hyp in the viral envelope could affect its fluidity,^36,37^ thus increasing the energy barriers to be overcome for viral-host membrane fusion.^2^ However, additional investigation is needed to better understand such mechanisms and their possible synergistic action.

## Conclusions

In this work, we demonstrated that Hyp binds to the membrane envelope of SARS-CoV-2 with a strong affinity (nanomolar *K*_D_) and estimated that a maximum of 30 Hyp can be loaded on a single viral particle, even if with marked heterogeneity among single viral particles. Moreover, we showed that Hyp induces a significant reduction of the viral infectivity toward Vero E06 cells, both upon light exposure and in the dark, suggesting the presence of multiple mechanisms of action.

More generally, these results highlight how the spectroscopic properties of Hyp can be successfully exploited for a quantitative investigation of its interaction with viruses, both on the ensemble and at the level of the single viral particles. This constitutes an essential requirement to outline the minimal conditions necessary to trigger the antiviral mechanisms of this drug, which is key in the development of more effective treatments. In this direction, further experiments will correlate the amount of loaded Hyp molecules with the induced photodamage on single viruses. Additionally, the effects of Hyp on the physicochemical properties of the virus (e.g., in terms of membrane fluidity or structural integrity) will be evaluated as potential complementary mechanisms of antiviral activity.

Finally, the observed interaction of Hyp with the envelope of SARS-CoV-2 has important implications for applications. On one hand, the nonspecific nature of Hyp binding to phospholipidic bilayers might be associated with a poor selectivity for SARS-CoV-2 (e.g., over mammalian cells), especially in systemic applications. On the other hand, it is noteworthy how Hyp binding and efficacy rely on rather general viral features, such as the presence of a membrane envelope. Thanks to this nonspecific action, Hyp and similar photosensitizing drugs are not subject to mutational pressure and are expected to effectively perform as broad-spectrum antivirals against a large variety of SARS-CoV-2 variants and of similar enveloped viruses. The strong antiviral activity observed against SARS-CoV-2 and the expected broad-spectrum action make Hyp an attractive photoantiviral agent, especially for localized treatments.

## Supporting Information Available

Additional spectroscopic data regarding the interaction of Hyp with viral particles, liposomes, and RBD proteins; electron microscopy images of SARS-CoV-2 particles; control experimental data. The Supporting Information is available free of charge at https://pubs.acs.org/doi/10.1021/acsami.1c22439.
