# Supplementary material for: The Interaction of Hypericin with SARS-CoV-2 Reveals a Multimodal Antiviral Activity
Source: ACS Appl Mater Interfaces. 2022 Mar 18;14(12):14025–32. doi: 10.1021/acsami.1c22439 (PMC8972258; doi:10.1021/acsami.1c22439)
Supplement: Supplementary file 1 — am1c22439_si_001.pdf [file am1c22439_si_001.pdf]

SUPPORTING INFORMATION

The interaction of hypericin with SARS-CoV-2 reveals a multimodal antiviral activity

Pietro Delcanale[1,*], Eleonora Uriati[1,2], Matteo Mariangeli[1,2], Andrea Mussini[1], Ana Moreno[3], Davide Lelli[3], Luigi Cavanna[4], Paolo Bianchini[2], Alberto Diaspro[2,5], Stefania Abbruzzetti[1,*], Cristiano Viappiani[1]

[1] Dipartimento di Scienze Matematiche, Fisiche e Informatiche, Università degli Studi di Parma, 43124 Parma, Italy
[2] Nanoscopy @ Istituto Italiano di Tecnologia, 16152 Genova, Italy
[3] Istituto Zooprofilattico Sperimentale della Lombardia e dell'Emilia Romagna, 25124 Brescia, Italy
[4] Dipartimento di Oncologia-Ematologia, Azienda USL di Piacenza, 29121 Piacenza, Italy
[5] DIFILAB, Dipartimento di Fisica, Università di Genova, 16146 Genova, Italy

*Corresponding authors:

Pietro Delcanale, e-mail: pietro.delcanale@unipr.it

Stefania Abbruzzetti, e-mail: stefania.abbruzzetti@unipr.it

ORCID

Pietro Delcanale: 0000-0001-8235-765X

Eleonora Uriati: 0000-0003-2894-8533

Matteo Mariangeli: 0000-0001-8069-6164

Andrea Mussini: 0000-0001-5363-7150

Ana Moreno: 0000-0002-8497-9708

Davide Lelli: 0000-0002-5775-6058

Luigi Cavanna: 0000-0002-0303-3778

Paolo Bianchini: 0000-0001-6457-751X

Alberto Diaspro: 0000-0002-4916-5928

Stefania Abbruzzetti: 0000-0001-7685-8554

Cristiano Viappiani: 0000-0001-7470-4770

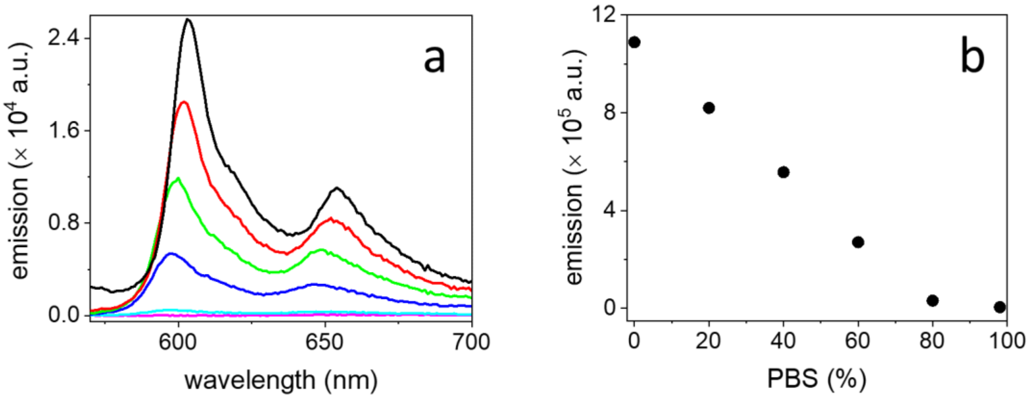

Figure S1. *(a) Fluorescence emission spectra obtained for 50 nM Hyp in different DMSO/PBS buffer mixtures: 98 % (magenta), 80 % (cyan), 60 % (blue), 40 % (green), 20 % (red) and 0 % (black) of PBS. The emission spectra reduce in intensity at increasing aqueous fraction of the mixture, while displaying the same shape and a slight shift towards shorter wavelengths. This is consistent with a fluorescence quenching caused by the hydrophobic aggregation of Hyp. (b) Integrated fluorescence emission obtained from the same spectra, at increasing PBS content. It is clearly visible how the fluorescence quenching scales with the amount of aqueous buffer in the DMSO/PBS mixture. When DMSO content is below 20 %, Hyp emission becomes negligible. Results are in agreement with previous literature.*[1–3]

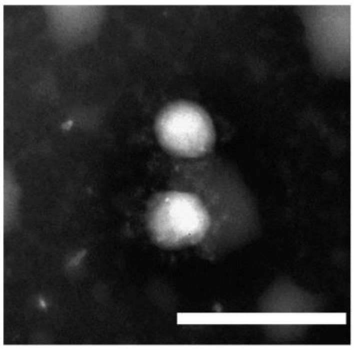

Figure S2. *Representative transmission electron microscopy image of the employed SARS-CoV-2 particles. The morphology of the viral particles is typical of coronaviruses: round particles with an external "corona" made of peplomers, with a size ranging between 85-90 nm to 110-120 nm (including peplomers). Scale bar 200 nm. Results are in agreement with previous literature.*[4]

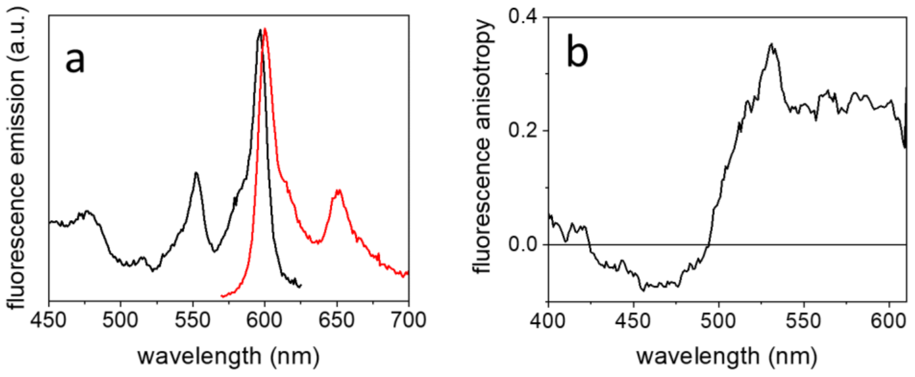

Figure S3. *(a) Excitation (black) and emission (red) spectra of 50 nM Hyp incubated with SARS-CoV-2 viral particles. (b) Fluorescence anisotropy spectrum of 50 nM Hyp incubated with ~1 nM SARS-CoV-2 viral particles. These results show that Hyp is solubilized in the presence of viruses, recovering its characteristic fluorescence emission. Non-zero anisotropy indicates that the rotation of emitting Hyp molecules is hindered due to the interaction with bulky viral particles.*

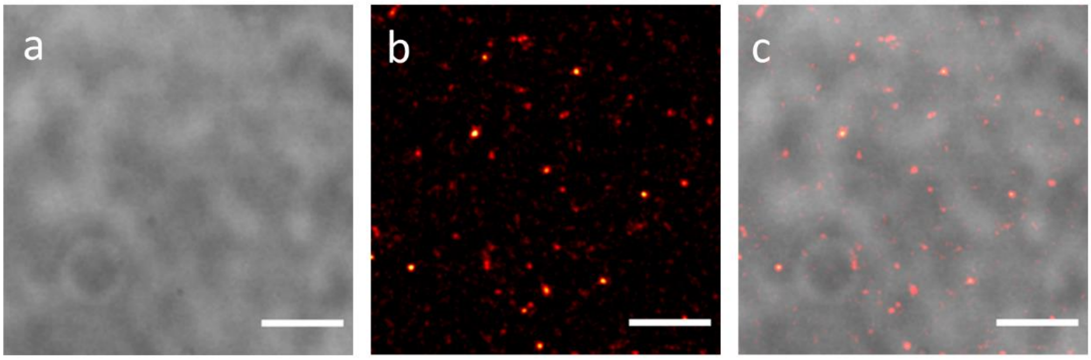

Figure S4. *(a-b) Bright field (a) and corresponding confocal fluorescence images (b) of SARS-CoV-2 particles exposed to 30 nM Hyp. (c) Overlap of the previous images. Scale bars 5 μm. It can be appreciated how viral particles cannot be recognized in the bright field image, because of the small size and the lack of contrast.*

[Hyp]	[SARS-CoV-2] (nM)	D (μm²/s)
2 nM	0.71	2.93
	0.51	1.15
	1.12	2.62
5 nM	0.47	1.7
	1.6	1.35
	0.89	2.61
	0.42	1.91
10 nM	0.64	0.6
	1.02	1.87
	0.33	1.86
20 nM	0.21	2.15
	0.65	-
	0.12	1.71
30 nM	0.18	1.71
40 nM	0.18	1.94
	2.1	0
	2.3	2.68
	1.8	2.31
	4.5	4.5
50 nM	0.33	1.46
	0.91	1.72
	0.22	1.82
70 nM	0.52	1.86
	0.44	2.3
	0.47	3.1
	0.6	2.2
90 nM	0.35	2.59
	9	3.5
	8.8	3.4
	(1.4 ± 1.0) nM	(2.4 ± 1.2) μm²/s

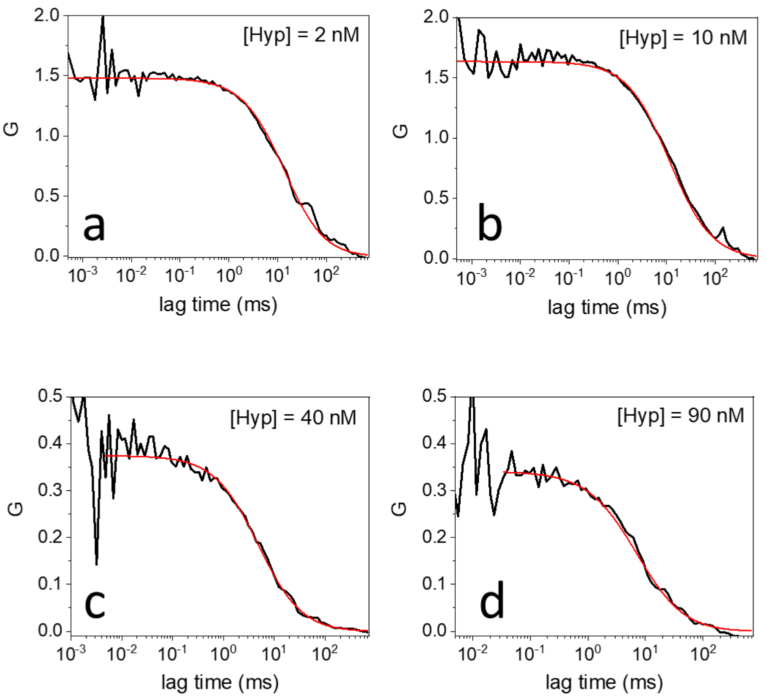

Figure S5. *The table shows the values of SARS-C0V-2 particles concentration and three dimensional diffusion coefficient (D) obtained from the fitting of the correlation curves measured by FCS. The resulting average values are highlighted in grey in the last row. (a-d) Examples of correlation curves measured by FCS (black) on SARS-CoV-2 particles exposed to 2 nM (a), 10 nM (b), 40 nM (c), 90 nM (d) Hyp. The red lines represent the results of a fitting with a model comprising a single diffusing species in solution. All data are acquired using the same dilution of viruses.*

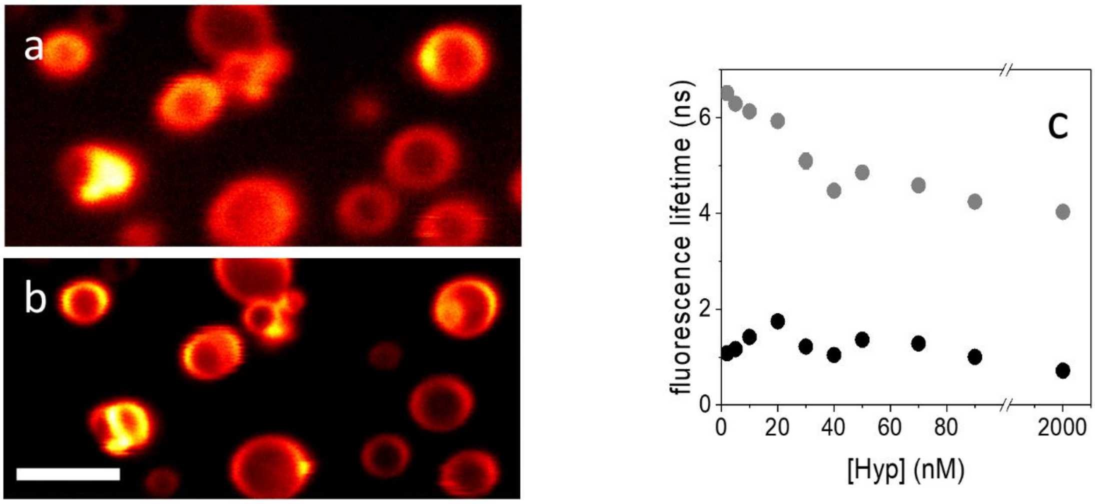

Figure S6. *(a-b) Confocal (a) and corresponding STED images (b) of Hyp incubated with DLPC liposomes. Scale bar 1 μm. The loading of Hyp on liposome membranes is clearly appreciated. (c) Fluorescence lifetimes, τ_1 (black) and τ_2 (grey), obtained from time-resolved fluorescence decays of Hyp, at increasing concentration, in the presence of the same amount of SARS-CoV-2 particles (~1 nM). Error bars are within circles. Each point was obtained from 3-5 repetitions. Lifetimes values are obtained by fitting the time-resolved fluorescence decays with a bi-exponential model.*

τ_2 values slightly decrease at increasing Hyp concentrations, until ~30 nM. This lifetime change likely originates from a partial self-quenching of Hyp, due to the fact that the virus-bound molecules are not perfectly isolated, but multiple molecules are simultaneously found on the same viral particle and might weakly interact, especially when approaching saturation. The lifetime values are more stable above ~30 nM Hyp, i.e. when viral particles are fully saturated. It should be noted that this weak quenching is not related to a strong hydrophobic aggregation, as happening for Hyp in aqueous media, and its effect on the overall intensity of fluorescence emission is marginal.

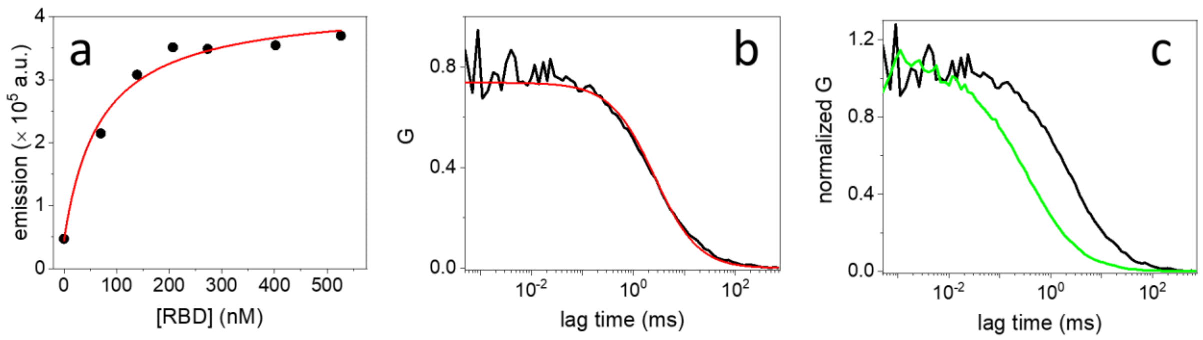

Figure S7. *(a) Integrated fluorescence emission of 50 nM Hyp at increasing concentration of recombinant RBD (black circles). The red line shows the result of the fitting with a binding model, that yielded an apparent equilibrium dissociation constant K_D = 60 nM. T = 23°C. (b) Representative correlation curve measured by FCS (black) on a solution containing an excess of RBD (80 nM, monomer) exposed to 10 nM Hyp. The red line shows the results of the fitting with a model comprising a single diffusing species. The three dimensional diffusion coefficient (D) obtained from the fitting is D ~ 5 $\mu m^2/s$, corresponding to diffusing species having a hydrodynamic diameter of ~100 nm. This diffusion (and size) is not consistent with that expected for Hyp bound to RBD monomers or small oligomers. (c) Comparison of normalized correlation curves measured by FCS obtained for RBD exposed to Hyp (black) and for RBD labeled with a streptavidin-conjugated Abberior STAR 635 fluorophore (green). The green curve may be regarded as a reference obtained for a solution containing diffusing species with a size comparable to that of RBD monomers or small oligomers. The much slower decay observed for Hyp exposed to RBD (black curve) indicates that Hyp is mainly bound to species having considerably slower diffusion, that very likely correspond to a fraction of large protein aggregates (~ 2 nM), in agreement with the results displayed in panel b.*

Panel a shows that, while aggregated Hyp in PBS buffer is virtually not fluorescent, its emission readily increases upon addition of RBD until a saturation value is reached. The recovery of Hyp emission in the presence of RBD indicates that the protein solubilizes Hyp aggregates and thereby suggests the existence of an interaction. However, it is essential to define if Hyp binds to the RBD monomer, which is the biologically relevant state. In panels b and c, FCS was used to measure the three-dimensional diffusion of protein-bound Hyp, for a solution containing Hyp and RBD. The results clearly show that Hyp is bound to a molecular species with a very slow diffusion (D = 2 - 9 $\mu m^2/s$, corresponding to hydrodynamic diameters of 50 – 150 nm). Such species do not correspond to RBD monomers or oligomers, which have a much faster diffusion, as shown in panel c. These results indicate that Hyp interacts with recombinant RBD organized into large aggregates, which do not correspond to the native biological state of the protein.

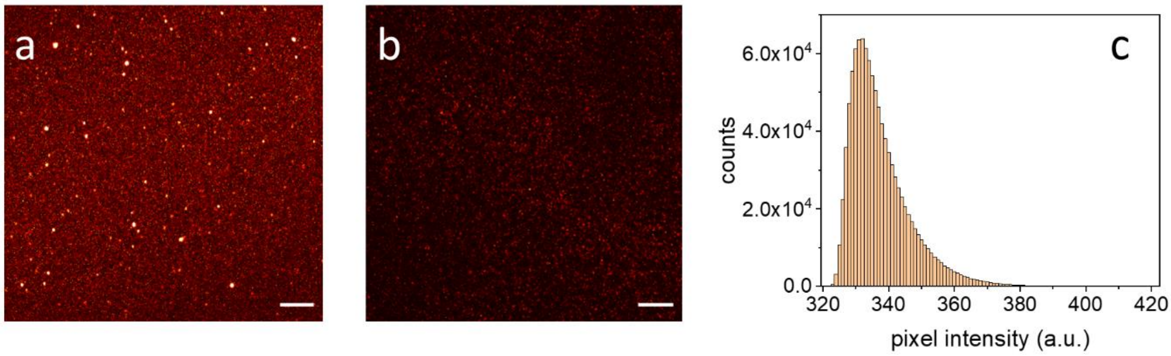

Figure S8. *(a) Representative image of SARS-CoV-2 particles exposed to 90 nM Hyp, acquired for the analysis of single particle intensity. (b) Corresponding control image acquired under the same conditions on a sample containing 90 nM Hyp, without SARS-CoV-2. Images are visualized with the same contrast. Scale bars 5 μM. (c) Pixel intensity distribution calculated from a control image obtained with 90 nM Hyp in absence of SARS-CoV-2. The image was acquired and processed under the same conditions employed for the analysis of single particle intensity. Pixel intensity never exceeds 400 a.u. The threshold values employed for particle selection during analysis are between 400 and 420 a.u., well above the background level measured in these controls.*

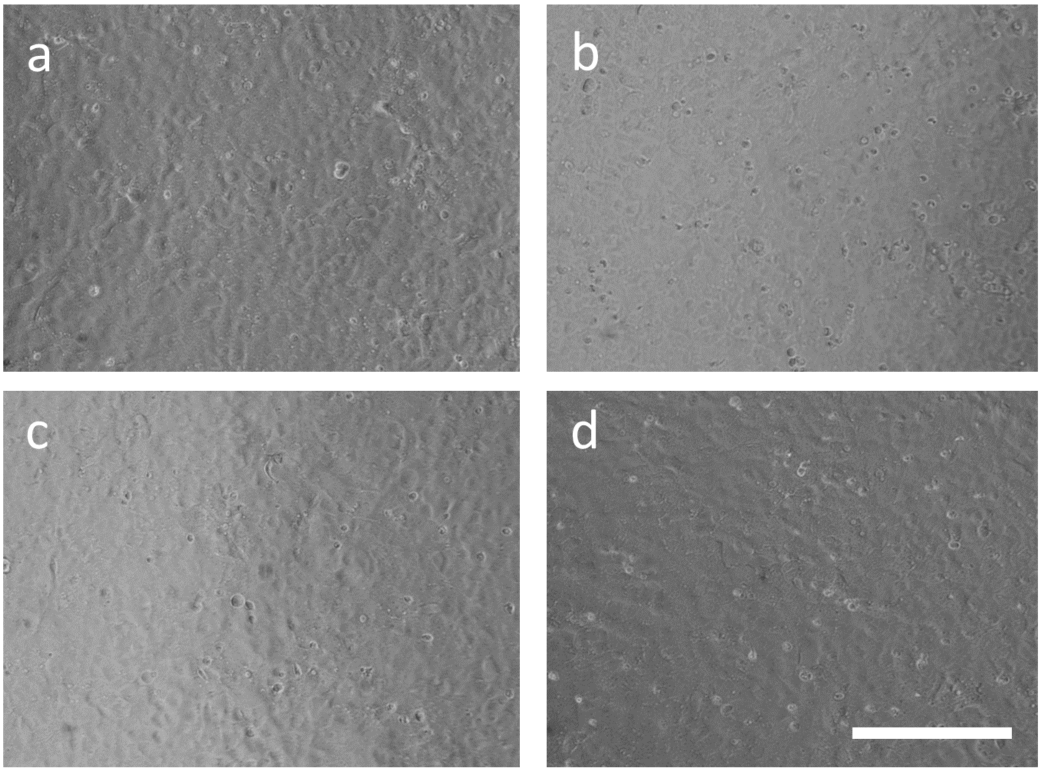

Figure S9. *Images of Vero E6 cells monolayers after exposure to control solutions (not containing viruses) that were either kept in the dark or irradiated with blue light prior to the administration to cells. (a-b) Vero E6 cells exposed to a solution containing only culture medium that was previously irradiated (a) or kept in the dark (b). (c-d) Vero E6 cells exposed to a control solution containing 300 nM Hyp that was previously irradiated (c) or kept in the dark (d). Scale bar 100 µm (all images). Experimental conditions were the same used in viral infectivity assays.*

All the control images show a perfectly intact and homogeneous cell monolayer. This demonstrates that, in the viral infectivity assays, cell viability is not directly affected by the presence of Hyp, either kept in the dark or previously irradiated.

(1) Bánó, G.; Staničová, J.; Jancura, D.; Marek, J.; Bánó, M.; Uličný, J.; Strejčková, A.; Miškovský, P. On the Diffusion of Hypericin in Dimethylsulfoxide/Water Mixtures—The Effect of Aggregation. *J. Phys. Chem. B* **2011**, *115* (10), 2417–2423. https://doi.org/10.1021/jp109661c.
(2) Delcanale, P.; Rodríguez-Amigo, B.; Juárez-Jiménez, J.; Luque, F. J.; Abbruzzetti, S.; Agut, M.; Nonell, S.; Viappiani, C. Tuning the Local Solvent Composition at a Drug Carrier Surface: The Effect of Dimethyl Sulfoxide/Water Mixture on the Photofunctional Properties of Hypericin–β-Lactoglobulin Complexes. *J. Mater. Chem. B* **2017**, *5* (8), 1633–1641. https://doi.org/10.1039/C7TB00081B.
(3) Chen, Y.; Fang, Y.; Gu, H.; Qiang, J.; Li, H.; Fan, J.; Cao, J.; Wang, F.; Lu, S.; Chen, X. Color-Tunable and ESIPT-Inspired Solid Fluorophores Based on Benzothiazole Derivatives: Aggregation-Induced Emission, Strong Solvatochromic Effect, and White Light Emission. *ACS Appl. Mater. Interfaces* **2020**, *12* (49), 55094–55106. https://doi.org/10.1021/acsami.0c16585.
(4) Ke, Z.; Oton, J.; Qu, K.; Cortese, M.; Zila, V.; McKeane, L.; Nakane, T.; Zivanov, J.; Neufeldt, C. J.; Cerikan, B.; Lu, J. M.; Peukes, J.; Xiong, X.; Kräusslich, H.-G.; Scheres, S. H. W.; Bartenschlager, R.; Briggs, J. A. G. Structures and Distributions of SARS-CoV-2 Spike Proteins on Intact Virions. *Nature* **2020**, *588* (7838), 498–502. https://doi.org/10.1038/s41586-020-2665-2.
